# Management of Cholelithiasis in Cirrhotic Patients

**DOI:** 10.3390/jpm12122060

**Published:** 2022-12-14

**Authors:** Viscosi Francesca, Fleres Francesco, Cucinotta Eugenio, Mazzeo Carmelo

**Affiliations:** Department of Human Pathology of the Adult and Evolutive Age “Gaetano Barresi”, Section of General Surgery, University of Messina, Via Consolare Valeria, 98125 Messina, Italy

**Keywords:** liver cirrhosis, Child–Pugh, cholelithiasis, cholecystectomy

## Abstract

Gallstone disease (GD) is a common disease worldwide and has a higher incidence in cirrhotic patients than in the general population. The main indications for cholecystectomy surgery in cirrhotic patients remain symptomatic cholelithiasis and its complications. Over the past two decades, numerous published reports have attested to the feasibility and safety of laparoscopic cholecystectomy in cirrhotic patients. Surgery in patients with liver cirrhosis represents an additional source of stress for an already impaired liver function and perioperative complications are remarkably high compared to non-cirrhotic patients, despite significant advances in surgical management. Therefore, preoperative risk stratification and adequate patient selection are mandatory to minimize postoperative complications. We have conducted a systematic review of the literature over the last 22 years for specific information on indications for surgery in cirrhotic patients and individual percentages of Child–Pugh grades undergoing treatment. There are very few reported cases of cholecystectomy and minimally invasive treatment, such as percutaneous transhepatic cholecystostomy (PTC), in patients with Child–Pugh grade C cirrhosis. With this work, we would like to pay attention to the treatment of cholelithiasis in cirrhotic patients who are still able to undergo cholecystectomy, thus also encouraging this type of intervention in cases of asymptomatic cholelithiasis in patients with Child–Pugh grades A and B.

## 1. Introduction

Gallstone disease (GD) is a common disease worldwide and, according to the European Association for the Study of the Liver (EASL), around 20% of Europeans are affected by this pathology [1]. It has been recognized that cholelithiasis has a higher incidence in cirrhotic patients than in the general population [2]. Cirrhosis is a condition of chronic liver function impairment, which unfortunately has systemic consequences in affected patients. There are several predisposing factors in cirrhotic individuals that contribute to the formation of gallstones and the consequent possibility of symptomatic cholelithiasis or acute cholecystitis, which represent the main indications for surgical treatment. Systemic complications and local anatomical consequences related to cirrhosis pose risks to both the anesthetist and the surgeon due to the perioperative complications. Therefore, the treatment of cholelithiasis and acute cholecystitis in cirrhotic patients has become a challenging problem. For this reason, in this review, we want to focus on the adequate therapeutic strategy, considering the risk stratification and the severity of liver cirrhosis.

## 2. Materials and Methods

**Figure 1:**
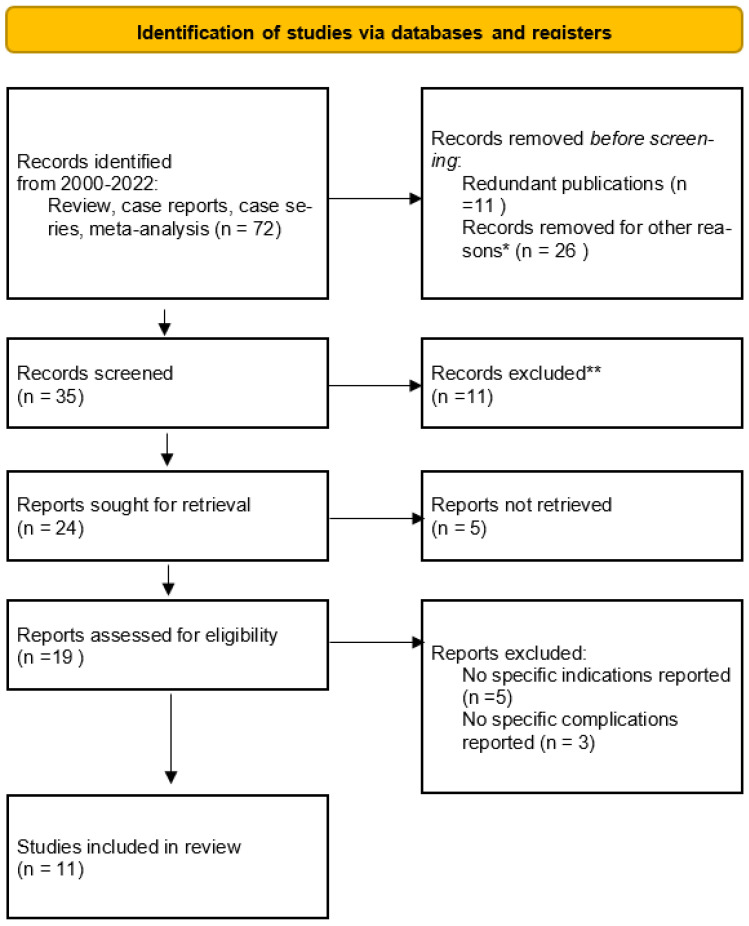
PRISMA 2020: Management of cholelithiasis in cirrhotic patients. * Child–Pugh degree of cirrhosis of the patients under examination was not specified. ** 11 papers were excluded by authors, duplicate records.

We conducted a systematic review of the literature over the last 22 years (2000 to 2022) using the keywords “Cirrhosis”, “cholecystectomy” and “indications”. Only studies published in English that detailed the Child–Pugh grade in the cirrhotic patients under review were considered. A total of 72 papers were analyzed, and, of these, the papers that were excluded were those that presented redundant publications, those in which the Child–Pugh degree of cirrhosis of the patients under examination was not specified, those in which the surgical indications were not specified and, finally, those in which postoperative complications were not specified. We analyzed 11 articles as defined by “PRISMA statement 2020” (Figure 1) [3,4,5,6,7,8,9,10,11,12,13,14]. These selected articles were reviewed for specific information on indications for surgery in cirrhotic patients and the individual percentages of Child–Pugh grades undergoing treatment (Table 1).

## 3. Results

From the literature analysis of the last 22 years, it is clear that the treatment of choice for symptomatic and/or complicated gallbladder stones is laparoscopic cholecystectomy, which has good outcomes in both patients with Child–Pugh grade A and B cirrhosis. There are always very few reported cases of cholecystectomy in patients with Child–Pugh grade C cirrhosis. Furthermore, percutaneous drainage of the gallbladder, carried out in the case of acute cholecystitis, must always be followed by cholecystectomy so that this pathology is completely resolved. The number of PTCs performed on Child–Pugh grade C cirrhotic patients is, as well, very few.

Table 1 shows the eleven articles analyzed in the literature, reporting the percentage of patients undergoing surgical treatment, based on the Child-Pugh classification.

**Table 1:** The actual indication for surgery in cirrhotic patients and the percentage by Child-Pugh grade undergoing treatment.

	Years	Tot	Child–Pugh Score			Indications	Treatment
			A	B	C		
**Quillin et al. [4]**	2000–2009	85	63 (74%)	20 (24%)	2 (2%)	Symptomatic cholelithiasis	Laparoscopic cholecystectomy
**McGillicuddy et al. [5]**	2002–2012	32	11 (34%)	14 (44%)	5 (16%)	Symptomatic cholelithiasis	Laparoscopic cholecystectomy
**Yao et al. [6]**	2004–2011	29	6 (21%)	19 (65%)	4 (14%)	Acute cholecystitis	PTC followed by a delayed LC
**Lledó et al. [7]**	2006–2010	43	26 (60%)	15 (35%)	2 (5%) Acute cholecystitis	Symptomatic cholelithiasis and Acute cholecystitis	Laparoscopic cholecystectomy
**El Nakeeb et al. [8]**	2008–2009	120	87 (72%)	33 (28%)	0	Symptomatic cholelithiasis	Laparoscopic cholecystectomy
**Târcoveanu et al. [9]**	2010–2020	111	82 (74.8%)	28 (25.3%)	1 (0.9%)	Symptomatic cholelithiasis and Acute cholecystitis (27.4%)	Laparoscopic cholecystectomy
**Gad et al. [10]**	2011–2019	213	127 (59.6%)	77 (36.2%)	9 (4.2%)	Acute cholecystitis (26.3%) Symptomatic cholelithiasis (68.1%) Pancreatitis (5.6%)	Laparoscopic cholecystectomy
**Bessa et al. [11]**	2011	40	27 (67%)	13 (33%)	0	Symptomatic cholelithiasis	Laparoscopic cholecystectomy
**Kassem et al. [12]**	2013–2016	62	35 (56.6%)	27 (43.5%)	0	Acute cholecystitis	Laparoscopic cholecystectomy Subtotal cholecystectomy (19.3%)
**Tan et al. [13]**	2013–2018	188	154 (82%)	28 (14%)	6 (4%)	Symptomatic cholelithiasis and Acute cholecystitis	Laparoscopic cholecystectomy
**Alhamid et al. [14]**	2014–2018	54	38 (72%)	12 (21%)	4 (8%)	Acute cholecystitis	Laparoscopic cholecystectomy

## 4. Discussion

Patients with liver cirrhosis are more likely than the general population to develop cholelithiasis. There are several pathogenic mechanisms that improve the formation of gallbladder stones: hypersplenism that causes chronic hemolysis, hypomotility of the gallbladder, reduced secretion of cholesterol, reduced synthesis of bile acids and their transportation. Any of these factors can influence the pathogenesis of gallstones and the onset of biliary colic, acute cholecystitis and biliary pancreatitis. Once these pathologies have developed, cholecystectomy surgery is mandatory. Surgical indications in cirrhotic patients have always been a debated topic. Due to the increased perioperative and postoperative complications in patients with liver cirrhosis, the surgical indications have always been very limited. In particular, cholecystectomy surgery in patients without cirrhosis of the liver is associated with a lower complication rate than in cirrhotic patients, due to several intrinsic risk factors of this pathology. The hardness of the fibrotic liver and the increase in vascularity secondary to portal hypertension are of considerable importance. Improved operating skills, equipment and accumulated experience in performing minimally invasive cholecystectomy under challenging conditions over the last years have made this surgery in cirrhotic patients a safe proposition when used with judgment. Before the introduction of laparoscopy, open cholecystectomy was the traditional treatment for symptomatic gallstones, with catastrophic postoperative complications. Thanks to the introduction of minimally invasive surgery, cholecystectomy has become an achievable procedure even for cirrhotic patients. Postoperative complications are mainly related to the Child–Pugh class, being maximal in Child–Pugh class C patients. Certainly, adequate patient selection, adequate preoperative optimization and appropriate use of instruments have led to lower morbidity and significantly lower mortality.

### 4.1. Risk Stratification

Mortality and morbidity rates of patients with liver cirrhosis undergoing non-hepatic abdominal surgery remain high [15]. Surgery represents an additional source of stress for these particular patients due to their impaired liver function and perioperative complications appear frequently, despite significant advances in surgical management and intensive care. Therefore, it is essential to consider preoperative risk stratification for cirrhotic patients in order to minimize postoperative complications. The risks of mortality and morbidity are related to the severity of the underlying liver cirrhosis. It is very often difficult to calculate preoperative risk stratification due to the limited accuracy of the tools available to evaluate a cirrhotic liver and, in some cases, the absence of a correct preoperative diagnosis. There are also other factors that may explain the wide variation in the results recorded by different studies in the literature, such as the different characteristics of the patient, the different surgical approaches adopted by surgeons and the varying levels of competence of surgeons, anesthetists and intensive care unit (ICU) staff. Portal hypertension (PHT) present in cirrhotic patients causes variceal bleeding, spontaneous bacterial peritonitis (SBP), hepatic encephalopathy and ascites. Therefore, patients with liver cirrhosis have an increased risk of liver complications during surgery due to the inadequate response to surgical stress.

Portal hypertension is not only an indicator of liver disease progression but increases perioperative mortality in patients undergoing non-hepatic abdominal surgery [16]. The presence of a high intraoperative PHT is associated with a higher mortality from postoperative rebleeding. Preoperative evaluation of liver function by a physician experienced in managing liver diseases is required for patients with suspected or diagnosed liver cirrhosis. In clinical practice, the gravity should be assessed by Child–Pugh (CP) and Model for- End-Stage Liver Disease (MELD) scores, although MELD is more objective [17]. The CP score is the most popular tool for preoperative risk stratification. Currò et al. reported a series of four patients with CP class C cirrhosis, with a morbidity of 75% and a mortality of 50% [18]. Another study [19] highlights the same results found in the literature, where there were no significant differences in mortality and morbidity in the results between Child–Pugh class A and B cirrhotic patients undergoing laparoscopic cholecystectomy. The MELD score, used primarily to prioritize the liver transplant waiting list, was recently introduced to predict the postoperative outcome of cirrhotic patients undergoing surgical procedures. However, there are still few scientific works in the literature that use this score in meta-analyses on cirrhotic patients undergoing cholecystectomy. Several studies in the literature have shown that a high MELD score correlates with a high rate of surgical complications. According to Dolejs et al. [20], in fact, the mortality of cirrhotic patients with MELD scores above 20 was 5.8%, while the morbidity was 22.8%. Fleming et al. also took ascites into account, coupled with the MELD score [21]. In his study, patients with MELD scores above 15 and ascites may have a mortality and morbidity of 23.53% and 47.06%, respectively, when undergoing laparoscopic procedures. Other laboratory parameters, more commonly used in clinical practice, have also been proposed to accurately predict postoperative morbidity after cholecystectomy in patients with cirrhosis. Perkins et al. [22] demonstrated that postoperative morbidity is significantly related to the patient’s coagulation pattern, in particular to the international normalized ratio, to preoperative platelet values, which in cirrhotic patients are decreased, to the increase in bilirubin and creatinine levels. These laboratory parameters are related to changes in liver function and underlying portal hypertension, although they can sometimes be altered due to gallbladder or gallstone disease.

### 4.2. Surgical Indications

In general, patients with asymptomatic cholelithiasis should not be treated. Medical therapy for the dissolution of bile acid is not decisive for symptomatic cholelithiasis, especially in cirrhotic patients, in whom the motility of the gallbladder is significantly reduced. Symptomatic cholelithiasis and complicated cholelithiasis (such as acute cholecystitis, obstruction of the biliary tract and pancreatitis), on the other hand, find indications for surgical, endoscopic or radiological treatment according to situations. Since its introduction in the 1980s, laparoscopic cholecystectomy (LC) has become the procedure of choice for cholelithiasis, and numerous studies have confirmed its safety in the general population. For patients with cirrhosis, on the other hand, this pathology was considered by surgeons an absolute or relative contraindication to laparoscopic cholecystectomy due to both the potential for bleeding and the onset of postoperative liver failure. Over the years, advances in surgical equipment have allowed for a gradual expansion of indications for laparoscopic cholecystectomy and made cirrhosis only a relative contraindication. In fact, in the last two decades, numerous scientific studies have documented the feasibility and safety of laparoscopic cholecystectomy in cirrhotic patients. These patients, who present greater operative risks in terms of bleeding and wound infections, can benefit even more from the minimally invasive approach. The open technique, in fact, can cause greater risks of bleeding and surgical wound infections. Most of these literature articles reported no significant increase in morbidity or mortality in patients with Child–Pugh class A or B cirrhosis undergoing laparoscopic cholecystectomy surgery. Unfortunately, however, there are still little data in the literature regarding patients with Child–Pugh class C cirrhosis.

The most frequent complication of cholelithiasis is acute cholecystitis. Treatment of acute cholecystitis consists of the administration of antibiotics and subsequent cholecystectomy. Cirrhotic patients with gallbladder stones complicated by acute cholecystitis will have a mostly unfavorable clinical course. In fact, two studies have shown that more than a third of patients with acute cholecystitis who have not undergone cholecystectomy surgery can develop complications such as biliary pancreatitis or choledocholithiasis within 2 years of the acute event [23,24]. Therefore, any patient with acute cholecystitis confirmed to be suitable for surgery should be subjected to early cholecystectomy after hospitalization to avoid subsequent complications. Even in the case of acute cholecystitis, the surgical gold standard remains the laparoscopic approach.

Treatment decisions for cirrhotic patients with acute cholecystitis, of any degree of severity, can be controversial. In fact, the severity of the inflammation is a limiting factor for immediate surgery in this group of patients, who still have unacceptably high rates of morbidity and mortality [25]. For cirrhotic patients, variceal vessels associated with portal hypertension and an easier bleeding tendency can interfere with surgery. If a good exposure of the Calot triangle cannot be obtained, laparoscopic subtotal cholecystectomy would be an alternative choice. For difficult gallbladders, such as severe cholecystitis, gallbladder empyema, perforated gallbladder, or the presence of marked portal hypertension, the surgeon may decide to perform a subtotal cholecystectomy, to avoid damage to blood vessels or to the bile duct. The subtotal cholecystectomy technique consists of opening the gallbladder at the level of the fundus or infundibulum, aspirating its contents, then resection and removal of the anterior wall; then, the posterior wall is removed (reconstructive technique) or coagulated and remains attached to the bed of the gallbladder (fenestration technique); the cyst is then sutured from the inside or clipped or tied to the remaining gallbladder stump. The long-term outcome of patients undergoing subtotal cholecystectomy, however, is associated with the presence of stones retained in the remains of the gallbladder. The rate of calculations withheld ranges from 4% to 15% [26]. Although most of the retained stones can be recovered from ERCP, some patients still need to undergo resurgery to complete cholecystectomy.

Bleeding is a major complication after laparoscopic cholecystectomy in patients with cirrhosis. Puggioni and Wong, in their 2003 meta-analysis, documented a postoperative hepatic bleeding rate of 4.3% in cirrhotic patients undergoing laparoscopic cholecystectomy, and 2.8% of the patients developed a postoperative intra-abdominal hematoma [27]. The use of the electrocoagulator near the hepatic hilum and especially the bile duct should be avoided, if possible, to prevent potential thermal damage. Most of the studies in the literature that report the surgical techniques used in laparoscopic cholecystectomy document the use of different hemostatic devices in addition to the traditional hook cauterization to dissect the gallbladder from the biliary tract [28]. This helped to minimize the risk of conversion due to bleeding not readily controlled laparoscopically and the inability to correctly recognize the anatomy. Many studies have shown that the ultrasonically activated scalpel (Harmonic) is an effective and safe tool for dissection and hemostasis in both open and laparoscopic surgical procedures. To date, the Harmonic scalpel is used in laparoscopic cholecystectomy for cystic artery division and liver bed dissection. Advances in the harmonic tip of the scalpel blade currently provide reliable ultrasonic cleavage and closure of the cystic duct. The lateral energy spread is minimal, and the risk of distant tissue damage is lower than with the electrocoagulator. According to El Nakeeb et al., the Harmonic provides shorter anesthesia and operator times, a lower incidence of gallbladder perforation and less intraoperative bleeding [8]. In addition to energy devices, other hemostatic adjuvants, such as oxidized regenerated cellulose and Floseal^®^ hemostatic matrix (Baxter International, Inc., Deerfield, IL, USA), can also be used in LC with a positive impact [29].

Percutaneous transhepatic cholecystostomy (PTC) is indicated in cirrhotic patients who are not medically fit for immediate cholecystectomy. This procedure can be performed both transhepatically and transperitoneally. However, these patients have a high risk of hemorrhage due to portal hypertension and sepsis due to ascites. For these reasons, percutaneous drainage is mostly performed by the transhepatic route due to the risk of the loss of bile in the peritoneum and unintentional damage to the hepatic flexure of the colon. This procedure reduces local inflammation, thus decreasing the degree of difficulty of the surgery (tenacious adhesions and high risk of bleeding) and allows time to optimize cirrhotic patients for definitive surgery. According to the study by Currò et al., Child–Pugh grade C cirrhosis patients with acute cholecystitis were able to benefit from alternative procedures, such as the placement of a percutaneous cholecystostomy tube; this technique has been considered for several years as the treatment of choice for relieving the symptoms of acute cholecystitis and reducing infectious complications due to its relatively low morbidity and increased safety [18]. The success of PTC readily relieves symptoms within a brief period. In the study reported by Yao et al., all patients undergoing PTC demonstrated clinical improvement within 3 days; this improvement helped these patients transition to surgery with acceptable risks [6]. However, the reported rates of acute cholecystitis recurrence after cholecystostomy in patients with gallstones may range from 25% to 30% [30]. It has been shown in the literature that PTC typically leads to multiple readmissions, extended hospital stays and higher hospital costs [31]. One study found an adverse event rate of 10–17% in patients undergoing PTC [32]. Vedachalam et al. concluded that the presence of cirrhosis appears to be a risk factor for initial PTC compared to initial cholecystectomy, especially for the failure to follow cholecystectomy [33]. In addition, PTC has poor longitudinal results, although it is more widely used in the healthcare of cirrhotic patients. To obviate the inherent risks of PTC, such as dislocation of the drainage into the peritoneum, causing infected ascites and worsening the patient’s prognosis, a further therapeutic option is reported in the literature, such as positioning the transpapillary cystic duct via endoscopic retrograde cholangiopancreatography (ERCP) [34]. This procedure can be performed in specialized centers and its purpose is to place a stent inside the cystic duct in order to detente the gallbladder, reducing both symptoms and local inflammation. Transpapillary endoscopic drainage may not be feasible due to both the acute angulation of the cystic duct and the presence of stones in the main duct. To overcome these limitations, James et al. introduced another minimally invasive option: the association of the percutaneous ultrasound-guided drainage of the gallbladder with the endoscopic drainage of the cystic duct (EUS-GBD) [35]. EUS-GBD is most indicated in patients with acute cholecystitis, the presence of a tortuous cystic duct or the presence of stones within the cystic duct. Unfortunately, there are still few studies regarding this technique, and most of the patients enrolled were suffering from cirrhosis of type A and B. This is due to the fact that patients with decompensated cirrhosis or Child–Pugh’s cirrhosis C had higher risks of bleeding and postprocedural infections.

From our literature review, we found that surgeons tend to operate predominantly on Child–Pugh grade A and B cirrhotic patients with symptomatic gallstones, acute cholecystitis or acute biliary pancreatitis. Patients with severe (C) liver cirrhosis, on the other hand, tend to be treated conservatively or by percutaneous biliary drainage, due to greater intraoperative and postoperative risks. These latter patients have little chance of having cholecystectomy surgery in their lifetime, thus increasing the chances of developing life-threatening complications. In conclusion, it would be useful for grade A and B cirrhotic patients to undergo cholecystectomy even if they have asymptomatic cholelithiasis, in order to prevent complications due to cholelithiasis when their cirrhosis is decompensated, and they will no longer be able to endure surgery. Although the literature recommends laparoscopic cholecystectomy as the treatment of choice in symptomatic cholelithiasis in patients with non-decompensated liver cirrhosis, we have found some limitations in most of the published studies: most of the studies were retrospective; most of the studies only enrolled patients with CTP classes A and B, thus excluding patients with grade C cirrhosis; the postoperative complications described were various, some reported bleeding complications, others biliary complications, others systemic complications and some papers did not specify the Child–Pugh grade of the enrolled patients.

## 5. Conclusions

In recent years, symptomatic gallbladder stones and their complications in cirrhotic patients are treated surgically by laparoscopic cholecystectomy with satisfactory outcomes in both Child–Pugh grade A and B patients. Grade C patients unfortunately have little chance of undergoing both radical surgery and minimally invasive treatment, such as PTC, due to complications related to the disease. It would be desirable nowadays that grade A and B cirrhotic patients, who are good candidates for surgery, undergo cholecystectomy even in the case of asymptomatic cholelithiasis, because, if their underlying disease was to worsen, they would lose the chance to undergo surgery.

## Abbreviation

Laparoscopic Cholecystectomy	LC
Percutaneous transhepatic cholecystostomy	PTC
Gallstone	GD
Portal hypertension	PHT
Child–Pugh	CP
Model for-End-Stage Liver Disease	MELD
